# Detection of Aflatoxigenic and Atoxigenic Mexican *Aspergillus* Strains by the Dichlorvos–Ammonia (DV–AM) Method

**DOI:** 10.3390/toxins10070263

**Published:** 2018-06-27

**Authors:** Masayo Kushiro, Hidemi Hatabayashi, Kimiko Yabe, Alexander Loladze

**Affiliations:** 1 Food Research Institute, National Agriculture and Food Research Organization (NARO), 2-1-12 Kannon-dai, Tsukuba-shi, Ibaraki 305-8642, Japan; hata-shhkyt0109@docomo.ne.jp; 2 Department of Environmental and Food Sciences, Faculty of Environmental and Information Sciences, Fukui University of Technology, 3-6-1 Gakuen, Fukui, Fukui 910-8505, Japan; yabek@fukui-ut.ac.jp; 3 International Maize and Wheat Improvement Center (CIMMYT), Apdo, Postal 6-641, Mexico City 06600, Mexico; A.Loladze@cgiar.org

**Keywords:** aflatoxin B_1_, aflatoxin B_2_, fungal strain

## Abstract

The dichlorvos–ammonia (DV–AM) method is a sensitive method for distinguishing aflatoxigenic fungi by detecting red (positive) colonies. In this study, the DV–AM method was applied for the isolation of aflatoxigenic and atoxigenic fungi from soil samples from a maize field in Mexico. In the first screening, we obtained two isolates from two soil subsamples of 20 independent samples and, in the second screening, we obtained two isolates from one subsample of these. Morphological and phylogenic analyses of the two isolates (MEX-A19-13, MEX-A19-2^nd^-5) indicated that they were *Aspergillus flavus* located in the *A. flavus* clade. Chemical analyses demonstrated that one isolate could produce B-type aflatoxins, while the other produced no aflatoxins. These results demonstrate that the DV–AM method is useful for the isolation of both aflatoxigenic and atoxigenic *Aspergilli*.

## 1. Introduction

**Figure 1:**
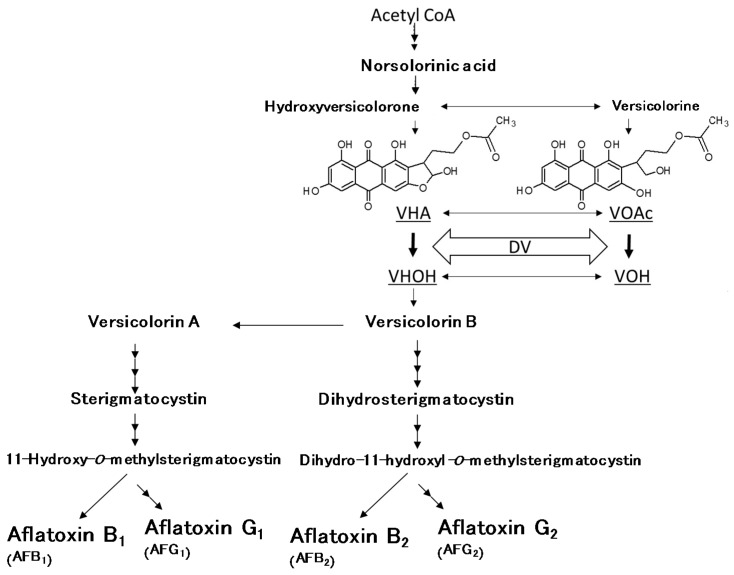
Outline of aflatoxin biosynthetic pathway and inhibition steps by dichlorvos (DV).

Aflatoxins (AFs) are mycotoxins produced mainly by certain strains of *Aspergillus flavus* Link and *Aspergillus parasiticus* Speare. Food and feed contamination with AFs is a global concern because they are highly toxic to animals and humans, causing acute hepatoxicity [1]. Eight AFs are biosynthesized in aflatoxigenic fungi through complex pathways [2,3,4] (Figure 1). Among four major AFs (aflatoxins B_1_ (AFB_1_), B_2_ (AFB_2_), G_1_ (AFG_1_), and G_2_ (AFG_2_)), AFB_1_ are classified as group 1 (human carcinogen) by the International Agency for Research on Cancer [5]. Mexico is a major maize-consuming country, and AF contamination is common. It is recommended that the sum of the four major AFs do not exceed 20 ppb in all foods [6].

We recently developed the dichlorvos–ammonia (DV–AM) method as a sensitive and simple approach to discriminate aflatoxigenic and atoxigenic fungi using several *A. parasiticus* strains [7]. In this method, fungi cultured on agar media supplemented with dichlorvos (DV) are treated by ammonia (AM) vapor to distinguish aflatoxigenicity visually. The underside of aflatoxigenic (positive) colonies shows exclusively a brilliant purple-red color, while atoxigenic (negative) colonies rarely change their color. The color change is triggered by the accumulation of versiconal hemiacetal acetate (VHA) and versiconol acetate (VOAc) via the inhibition of esterase by DV (Figure 1) [8]. The natural yellowish color of the VHA and VOAc compounds changes drastically under alkaline conditions because of the presence of the anthraquinone moiety in their chemical structures.

Aflatoxigenic *Aspergilli* are believed to be distributed mainly in tropical and subtropical regions [9,10]. Field soils with aflatoxigenic *Aspergilli* are considered as an original source of AF contamination in agricultural products after harvest. A high frequency of aflatoxigenic *Aspergilli* in field soil has been previously reported [11,12,13]. The contamination with AFs in cereals and commodities such as maize, nuts, cottonseed, spices, and dried fruit is known [14]. Maize is the most frequently reported cereal with AF contamination, and a number of people have died reportedly from the consumption of maize contaminated with AFs [15].

Various pre- and post-harvest genetic approaches, chemical, and cultural control methods are practiced to minimize or prevent AF contamination in maize [16,17,18,19,20,21]. These include breeding for improved resistance to pre-harvest AF accumulation, control of *Aspergilli* in the field with fungicides, and control of pest insects (vectors for *Aspergillus* spp.) with improved host resistance via transgenic approaches or insecticides. In addition, several indirect control methods, such as adjusting planting dates and planting densities, optimizing irrigation practices, improving soil fertility, etc. are utilized to combat the fungi. These cultural practices mainly aim at reducing abiotic stresses to the plants (e.g., drought, nutrient deficiencies), since *Aspergilli,* being opportunistic parasites, infect and colonize easily weakened and stressed plants.

Biological control methods, which utilize atoxigenic strains of various *Aspergilli* to suppress the toxigenic strains of the same species, have been gaining more attention. Trials to control AF production and contamination in the field using atoxigenic *Aspergilli* started from 1990s [22,23,24]. The biological control of AF production using atoxigenic *A. flavus* strains (Aflasafe KE01^TM^) has proven to be effective in Kenyan maize fields.

To detect and discriminate aflatoxigenic and atoxigenic *Aspergilli*, a number of methods have been developed [25]. Molecular marker-based methods (using specific gene regions) and fungal culturing methods (using characteristic secondary metabolites) are two major strategies for fungal discrimination. Presently, the molecular marker-based methods are not yet very reliable compared to the fungal culturing methods, since the presence of specific gene regions related to AF biosynthesis does not always confirm the aflatoxigenicity of some strains because of mutations that are common in those gene clusters [26]. Currently, in most cases, aflatoxigenic fungi are being identified by the *A. flavus* and *parasiticus* agar (AFPA) method using ferric chloride on agar media [27]. Occasionally, the AFPA method provides false-positive results for AF production because it detects both aspergillic and noraspergillic acids [27]. These are common metabolites of two major aflatoxigenic species (*A. flavus* and *A. parasiticus*) but are not related to AF biosynthesis and metabolism [28]. In contrast, the DV–AM method detects biosynthetic intermediates of AFs [29]. The objective of the current study was to examine the efficiency of the DV–AM method for the detection and identification of aflatoxigenic and atoxigenic *Aspergilli* in soil samples from Mexico.

## 2. Results

### 2.1. Isolation of Aspergilli from Soil Subsamples

**Figure 2:**
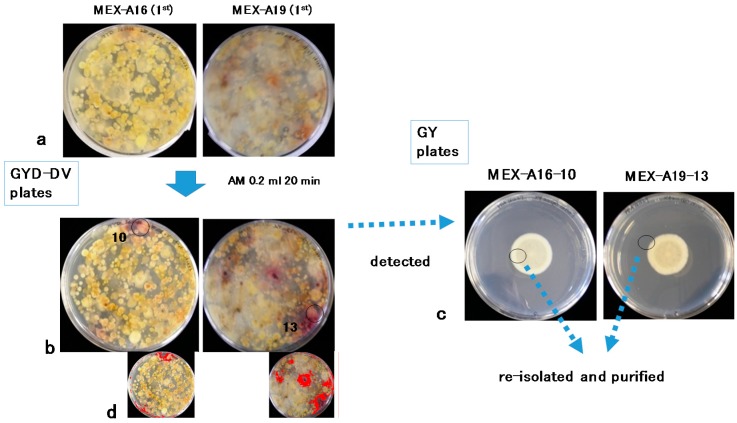
(**a**) Dichlorvos–ammonia (DV–AM) method (1st screening) before ammonia (AM) treatment; (**b**) After AM treatment; (**c**) After detection; (**d**) After image-processing with ImageJ software.

**Figure 3:**
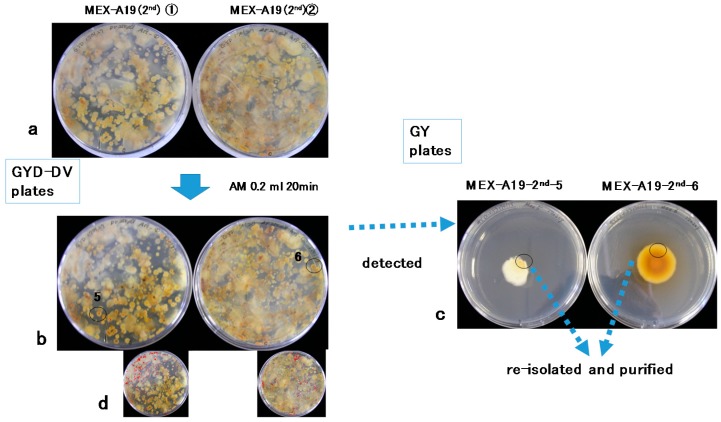
(**a**) DV–AM method (2nd screening) before AM treatment; (**b**) After AM treatment; (**c**) After detection; (**d**) After image-processing with ImageJ software.

A total of 20 soil sub-samples (A1–A20) cultured on glucose–yeast extract–sodium deoxycholate (GYD)–DV agar plates were treated with AM vapor. Among the observed *Aspergilli*, two colonies positive for aflatoxin production were detected on two subsamples (A16 and A19) in the first screening (MEX-A16-10 and MEX-A19-13) (Figure 2). The second screening was conducted using subsamples of A19 (Figure 3), and two additional isolates (MEX-A19-2^nd^-5 and MEX-A19-2^nd^-6) were obtained. The isolates MEX-A19-2^nd^-5 and MEX-A19-2^nd^-6 showed red color only in the center of each colony (Figure 3). In total, four isolates were obtained and were later purified for further analysis (Figure 2 and Figure 3).

Image processing to enhance red color intensity was conducted for the images of the first (Figure 2b) and the second screenings (Figure 3b) to generate the images presented in Figure 2d and Figure 3d, respectively. The isolates indicated by circles in MEX-A16-10 and MEX-A19-13 (Figure 2b, left and right, colony size; ca. 8 mm) were included in the red regions emphasized in the processed images (Figure 2d, left and right). On the other hand, the isolates indicated by circles of MEX-A19-2^nd^-5 and MEX-A19-2^nd^-6 (Figure 3b, left and right, colony size; ca. 4 mm) were outside of the red regions highlighted in the processed images (Figure 3d, left and right).

### 2.2. Determination of AFs 

**Figure 4:**
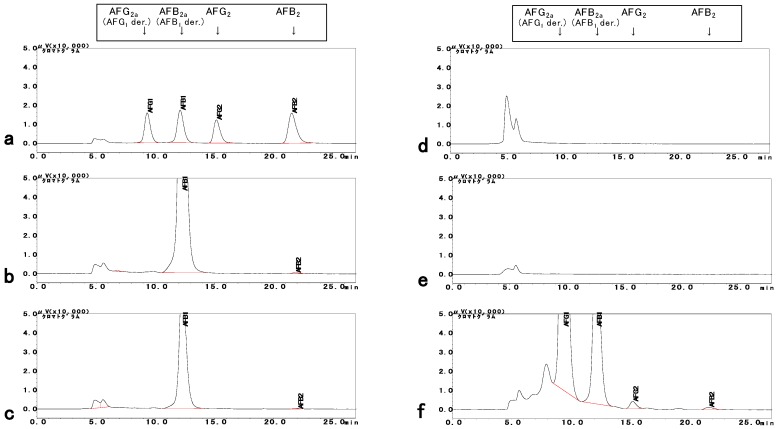
(**a**) Chromatograms of standard solution of aflatoxins (AFs) (10 ng/mL each); (**b**) Methanol extract of *Aspergilli* isolates MEX-A16-10; (**c**) MEX-A19-13; (**d**) MEX-A19-2^nd^-5; (**e**) MEX-A19-2^nd^-6; (**f**) OKI-12.

The four isolates were additionally purified three times. The purified isolates were cultured, scratched, and extracted with methanol [30]. The accumulation of AFs in the methanol extract was tested by HPLC–FL analysis (Figure 4). Both MEX-A16-10 and MEX-A19-13 (two isolates from the first screening) produced significant amounts of AFB_1_ (over 1000 ng/g) and small amounts of AFB_2_ (30–40 ng/g), while none of them produced G-type AFs (Figure 4b,c). On the other hand, neither MEX-A19-2^nd^-5 nor MEX-A19-2^nd^-6 (the two isolates from the second screening) produced any AFs (Figure 4d,e). A positive control strain, OKI-12, produced all four AFs in the same culturing conditions (Figure 4f). Therefore, the isolates MEX-A16-10 and MEX-A19-13 were confirmed to be aflatoxigenic (exclusive for B-type AFs), while MEX-A19-2^nd^-5 and MEX-A19-2^nd^-6 were classified as atoxigenic.

### 2.3. Phylogenetic Analysis of Isolates

**Figure 5:**
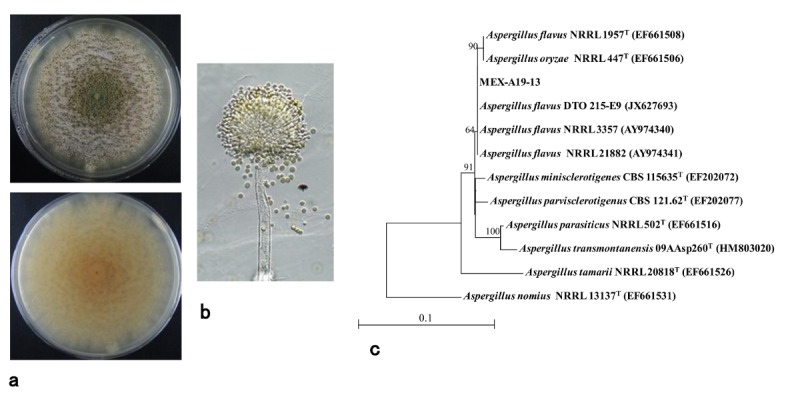
Properties of an *Aspergillus flavus* isolate MEX-A19-13: (**a**) Giant colony formed on potato dextrose agar (PDA) medium observed from the surface (top) and underside (bottom) of the culture; (**b**) Conidiophore and conidia; (**c**) Phylogenetic tree analysis based on calmodulin (*cmd*) gene sequence.

**Figure 6:**
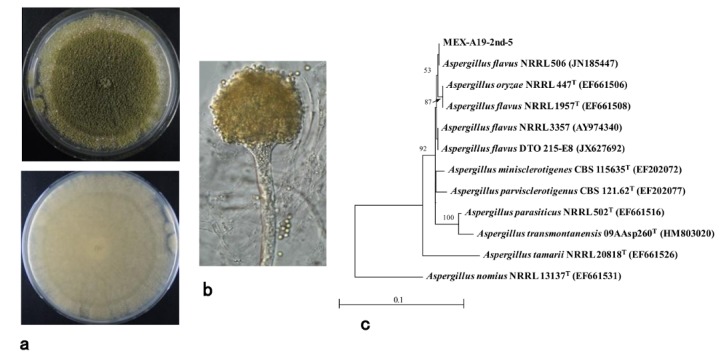
Properties of the *A. flavus* isolate MEX-A19-2^nd^-5: (**a**) Giant colony formed on PDA observed from the surface (top) and underside (bottom) of the culture; (**b**) Conidiophore and conidia; (**c**) Phylogenetic tree analysis based on *cmd* gene sequence.

The isolates MEX-A19-13 (aflatoxigenic; Figure 4c) and MEX-A19-2^nd^-5 (atoxigenic; Figure 4d) were further cultured for detailed morphological and phylogenetic analyses. These two purified isolates showed similar macroscopic features on glucose–yeast extract (GY) media (Figure 2c right and Figure 3c left). While the morphological features were different on potato dextrose agar (PDA) medium (Figure 5a and Figure 6a). Furthermore, a microscopic observation revealed that both isolates had the conidiophores and conidia morphologically typical for *Aspergilli* (Figure 5b and Figure 6b).

The sequences of the calmodulin (*cmd*) genes of the two isolates were used to determine the species. The *cmd* gene sequences of MEX-A19-13 and MEX-A19-2^nd^-5 were highly similar. The *A. flavus* strain HA9-S1-1, previously isolated from a sorghum field in Japan, also showed high similarity to these isolates (data not shown). The phylogenetic tree analysis suggested that these isolates were *A. flavus* located on *A. flavus* clade (Figure 5c and Figure 6c).

## 3. Discussion

The AF-positive isolates detected in the current study (MEX-A16-10 and MEX-A19-13) showed similar morphological and chemical properties. They were confirmed to belong to an aflatoxigenic *A. flavus* strain producing B-type AFs. This was further confirmed with the comparison of the *cmd* gene sequences of these isolates with those of *A. flavus* strains.

Despite having distinct morphological features, the other two isolates that did not produce AFs (MEX-A19-2^nd^-5 and MEX-A19-2^nd^-6) were also confirmed to belong to a strain of *A. flavus.* However, in contrast with the previous two isolates, they were classified as atoxigenic.

Certain *A. flavus* strains produce the B-type AFs, while others are atoxigenic. The strain MEX-A19-13 is of the former type (Figure 5) while MEX-A19-2^nd^-5 belongs to the latter type (Figure 6). This study showed that the DV–AM method was effective for the isolation of both types of *A. flavus*. The DV–AM method was originally developed for the simple visual detection and screening of aflatoxigenic *A. parasiticus* strains [7]. Previous studies revealed that the DV–AM method was applicable for the screening of aflatoxigenic *A. flavus* strains as well as of minor aflatoxigenic species such as *Aspergillus pseudonomius* [30,31]. However, until now, atoxigenic *Aspergilli* have not been detected by the DV–AM method. In this study, we concentrated on the small red colonies (colony size ca 4 mm) which were previously neglected. As a result, we were able to isolate successfully two atoxigenic *Aspergilli* colonies (Figure 3b).

The image processing to enhance the red color failed to detect atoxigenic isolates; therefore, detailed visual observation was crucial (Figure 3b,d). On the other hand, the DV–AM method combined with image processing may facilitate the screening of aflatoxigenic isolates, as demonstrated in Figure 2b,d. In this study, we did not compare the efficiency of screening of aflatoxigenic and atoxigenic isolates between the DV–AM method and other methods such as the AFPA method. However, we assumed that the DV–AM method was more effective to identify atoxigenic isolates in the case they were natural occurring variants with low accumulation of anthraquinone (the skeleton of VHA and VOAc)-related compounds. In such cases, a weak or limited color change of the related compounds in a small colony would occur, as shown in two colonies in Figure 3b. The compounds which may display a similar color change as VHA and VOAc in atoxigenic isolates should be clarified in further studies.

One of the aims of the current study was to apply the DV–AM method, which was originally developed to detect Japanese *Aspergilli*, to isolates of Mexican origin. Although the original technique was slightly modified with image enhancement, the method proved to be reliable and applicable to fungal isolates of diverse origins. Despite the relatively small number of samples used in the current work, the method was empirically proved to be functional and efficient. However, to assess the robustness of the method, a larger number of *Aspergillus* isolates, perhaps with wider and more diverse geographic origins, needs to be tested. This, in turn, may warrant further modifications, adjustments, and improvements of the method. In addition, a comparison of the efficiency of different diagnostic methods will be performed.

## 4. Conclusions

In this study, we successfully demonstrated the use of the DV–AM method for the detection of two types of strains with distinct AF production properties. Since the discrimination between aflatoxigenic and atoxigenic isolates is important, the method described in this study will be useful for isolate identification in the future. The criteria of colony size and red radii, shown in Figure 2b and Figure 3b, to distinguish aflatoxigenic and atoxigenic strains, will be developed further in the future studies.

## 5. Materials and Methods

### 5.1. Media 

For the isolation of *Aspergilli* fungi from soil samples, GYD agar medium (2% glucose, 0.5% yeast extract, 0.05% sodium deoxycholate, and 2% agar) was used. Sodium deoxycholate was supplemented to obtain compact colonies. For the confirmation of AF production, GY agar medium (2% glucose, 0.5% yeast extract, and 2% agar) was used. Potato dextrose agar medium (PDA; BD Difco, Detroit, MI, USA) was used for the morphological characterization.

### 5.2. Strains Used

A Japanese strain of *A. pseudonomius*, OKI-12 (MAFF 111900, Genebank, MAFF, Tsukuba, Japan) isolated from a sugarcane field in 2017, was used as a positive control for B- and G-group AFs production [30]. A Japanese strain of *A. flavus*, HA9-S1-1 (MAFF 111859, Genebank, MAFF, Japan), isolated from a sorghum field in 2015, was used as a reference for the phylogenetic analysis [31]. Within the current study, we isolated two aflatoxigenic and two non-aflatoxigenic strains, and these strains were named using the prefix MEX-A (MEX-A16-10 and MEX-A19-13, and MEX-A19-2^nd^-5 and MEX-A19-2^nd^-6, correspondingly).

### 5.3. Soil Sampling

Sampling was conducted in the field of a maize trial at the Agua Fria Experimental Station of CIMMYT in the state of Puebla, Mexico, (20.45° N, 97.64° W), at an altitude of 110 masl. The average yearly precipitation at the location is approximately 1200 mm, with the air temperature ranging from 5 to 42 °C, average relative humidity of 85%, and clay loam soils of pH 7.5 to 8.5. A total of 20 soil samples (A1–A20) (about 50 g each) were collected from a maize field on 13 July 2016 and kept at 4 °C until used. Sampling was done with a disinfected spatula, 2–3 cm underneath the soil surface and 3–4 cm away from maize plant roots.

### 5.4. DV–AM Method

A solution of 50 μL of 250-fold diluted dichlorvos (DV; Wako, Osaka, Japan) methanol was spread onto a 9 cm-diameter GYD agar plate and left for approximately 30 min to allow the solution to diffuse into the agar. Approximately 0.3 g of each soil sample was suspended in 1 mL of 0.05% Tween 80 aqueous solution, and 50 μL of this suspension was poured onto a GYD agar plate using a wide bore pipette tip, spread, and incubated at 25 °C in darkness for 3–5 d. Then, 0.2 mL of ammonium hydroxide solution (AM; 25–28%, analytical grade, Wako, Osaka, Japan) was poured onto the inside of the lid of the Petri plate. The bottom of the Petri plate was immediately placed back on top of the lid to allow the AM vapor to disperse. The treated Petri plate was kept inverted at room temperature for approximately 20 min. Color change was observed from the underside of the plates. The conidia of candidate colonies were picked out using a sterile toothpick and transferred to GY plates. The conidia had to be immediately transferred after AM treatment because a longer exposure to AM vapors was lethal to the fungal conidia. The resulting fungal isolates were further purified by three repetitions of single conidial isolation.

### 5.5. AF Production Analysis

To measure the AF production of the isolates, the agar scratch method was used [30]. The isolates transferred to GY agar media were harvested after 3–5 d of culturing and killed by exposure to AM vapor. Approximately 2–4 g of scratched GY with fungi were put into 50 mL centrifuge tubes and extracted with 5 mL of 100% methanol. For the HPLC analyses, 0.05 mL of the extract was dried, and 0.1 mL of trifluoroacetic acid (TFA) was added to convert AFB_1_/G_1_ into the highly fluorescent hemiacetal AFB_2_a/G_2_a. The TFA derivatization reaction was stopped after 15 min by the addition of 0.9 mL of 10% acetonitrile, and 0.005 mL of the resultant solution was injected into the HPLC system and analyzed using an LC column (0.3 × 25 cm, CAPCELL PAK C18, UG120) (Shiseido, Tokyo, Japan) and a mobile phase of water–acetonitrile–methanol (1:3:6, *v*/*v*/*v*). AF detection was performed by a fluorescence detector (excitation at 365 nm, emission at 450 nm), and the limit of detection for AFG_1_, AFB_1_, AFG_2_, AFB_2_ was 1.1, 0.93, 0.49, 0.58 ng/mL, respectively.

### 5.6. Identification of Fungal Species

The species identification of two isolates (an aflatoxigenic isolate MEX-A19-13 and an atoxigenic isolate MEX-A19-2^nd^-5) was conducted at Techno Suruga Laboratory (Shizuoka, Japan). Genomic DNA was extracted, and the genome sequences of the *cmd* genes were analyzed, followed by PCR amplification with CMD5 and CMD6 primers [32]. The resulting PCR products were sequenced using an ABI PRISM 3130xl Genetic Analyzer System (Applied Biosystems, Foster City, CA, USA). The obtained sequence data ere compared with those from DNA Data Bank of Japan (DDBJ) by using the BLAST search. The phylogenetic tree analysis was conducted using Aporon 3.0 (Techno-Suruga Lab., Shizuoka, Japan), on the basis of the neighbor-joining method of *cmd* gene sequence data.

The genomic nucleotide sequence data for the *cmd* genes of MEX-A19-13 and MEX-A19-2^nd^-5 were deposited in the DDBJ/ European Molecular Biology Laboratory (EMBL)/GenBank nucleotide sequence database under the accession nos. LC383381 and LC383382.

## Abbreviations

DV-AM	dichlorvos-ammonia
AF	aflatoxin
DV	dichlorvos
VOAc	versiconol acetate
VHA	versiconal hemiacetal acetate
AFPA	*Aspergillus flavus* and *parasiticus* agar
TFA	trifluoroacetic acid
HPLC	High-performance liquid chromatography
DNA	deoxyribonucleic acid
PCR	polymerase chain reaction
DDBJ	DNA Data Bank of Japan
EMBL	European Molecular Biology Laboratory
